# Effect of cartilaginous endplates on extruded disc resorption in lumbar disc herniation

**DOI:** 10.1371/journal.pone.0195946

**Published:** 2018-04-17

**Authors:** Kenichi Kawaguchi, Katsumi Harimaya, Yoshihiro Matsumoto, Mitsumasa Hayashida, Seiji Okada, Keiichiro Iida, Go Kato, Kuniyoshi Tsuchiya, Toshio Doi, Yoshinao Oda, Yukihide Iwamoto, Yasuharu Nakashima

**Affiliations:** 1 Department of Orthopaedic Surgery, Graduate School of Medical Sciences, Kyushu University, Fukuoka, Japan; 2 Department of Orthopaedic Surgery, Saga-Ken Medical Center, Saga, Japan; 3 Department of Orthopaedic Surgery, JCHO Kyushu Hospital, Fukuoka, Japan; 4 Department of Orthopaedic Surgery, Kyushu University Beppu Hospital, Oita, Japan; 5 Department of Anatomic Pathology, Pathological Sciences, Graduate School of Medical Sciences, Kyushu University, Fukuoka, Japan; University of Crete, GREECE

## Abstract

**Objective:**

The aim of this study was to investigate the clinicopathologic features of lumbar disc herniation (LDH) with endplate degeneration and the association between cartilaginous fragments and inflammatory response to the herniated disc.

**Summary of background data:**

LDH often involves hyaline cartilage fragments pulled from the vertebral endplates. Modic changes are closely associated with LDH that contains hyaline cartilage, and cartilaginous endplates seem to affect resorption of the herniated disc.

**Methods:**

A total of 78 patients who underwent microscopic discectomy between 9 and 16 weeks after an occurrence of LDH were reviewed. Modic changes, disc degeneration, high-intensity zone, and vertebral corner defect were evaluated using magnetic resonance imaging (MRI). Histopathological observations of cartilaginous endplates and inflamed granulation tissue in the herniated disc were made. In cases with inflamed granulation tissue, neovascularization and macrophage infiltration were also evaluated using immunohistochemical analysis.

**Results:**

Modic changes were observed in approximately one-third of the patients (26 cases: type 1, 7; type 2, 17; and type 3, 2). Cartilaginous endplates were observed in 32 cases (41%) and in the majority of cases with Modic changes compared with cases without Modic changes (65%, *p* = 0.001). Although inflamed granulation tissue was observed in 60 cases (77%), no significant differences were detected in patient age and the composition of the herniated material. Immunohistochemical analysis showed that fewer CD34-positive capillaries and CD68-positive cells were found in cases with cartilaginous fragments compared with those without cartilaginous fragments (*p* < 0.001). In addition, a higher immunoreactivity to CD34 and CD68 was found in herniated discs <25% of whose area was occupied by cartilaginous endplates compared with discs whose area was occupied at 25% or more (*p* < 0.001).

**Conclusion:**

There is an association between LDH with endplate degeneration and cartilaginous herniation, with Modic type 2 predominating. Furthermore, neovascularization and macrophage infiltration, especially if the amount of cartilage is high, are likely to be less frequent in cartilaginous herniation, leading to failure in the spontaneous remission of clinical symptoms.

## Introduction

Previous studies have reported that lumbar disc herniation (LDH) specimens contain varying populations of the nucleus pulposus, annulus fibrosus, cartilaginous endplates, and bone tissues [1–4]. The histological composition of the herniated disc fragments affects pain and clinical symptoms, and the presence of cartilage fragments is more likely in patients who report persistent sciatica [5,6]. Therefore, it is important to evaluate the histological composition of herniated discs, especially the amount of hyaline cartilage. Modic changes, which are present as signal alterations in the vertebral endplate and adjacent bone marrow, are found on T1- and T2-weighted magnetic resonance imaging (MRI); these conditions are associated with vertebral endplate fissures. Modic changes are known to be associated with LDH-containing cartilaginous fragments [3,7,8]. Schmid et al. reported the presence of a cartilaginous endplate in the extruded disc material in 63% of patients with Modic changes [3].

Some symptoms of LDH are benign in many cases because of the spontaneous resorption of some herniated discs [9–12]. The inflammatory response that occurs around the herniated tissue in the epidural space is believed to play an important role in herniated disc resorption as well [13,14]. Histologically, herniated material is surrounded by granulation tissue and is characterized by inflammatory cell infiltration and newly formed vessels [15–18]. Several studies suggest that the presence of a cartilaginous endplate may influence herniated disc resorption [5–7]. Hyaline components are known to have dense networks of collagen fibrils, resulting in the swelling of tissues and the loss of proteoglycan and subsequent resorption [5,19,20]. Shan et al. found that more capillaries and abundant macrophage infiltrates are observed in LDH without Modic changes than in LDH with Modic changes, which may indicate cartilaginous herniation that resorbs poorly [7]. However, less is known about the effect of cartilaginous endplates in LDH, and the association between the amount of cartilage endplate and spontaneous resorption has not been specifically investigated.

The purpose of this study was to (i) evaluate the clinicopathological characteristics of LDH with endplate degeneration and (ii) investigate the relationship between cartilaginous fragments and the inflammatory response of the herniated disc.

## Materials and methods

### Patients

One hundred and twenty-six patients with LDH for whom 8 weeks of conservative treatment failed were surgically treated by single-level microscopic discectomy from January 2013 to December 2016. The inclusion criteria were as follows: LDH on MRI with corresponding radicular pain, LDH with transligamentous extrusion and sequestration based on the classification of McCulloch and Macnab [21], and patients with disease duration between 9 and 16 weeks after an occurrence of LDH. The extrusion criteria were coexisting lumbar spinal canal stenosis or a history of lumbar surgery. Consequently, a total of 78 patients (49 males and 29 females) were enrolled in the present study. This study was approved by the Institutional Review Board of Saga-Ken Medical Center Koseikan prior to the start of data collection. The details of this study were explained to the patients, and all patients provided informed consent. Age at the time of surgery ranged from 21 to 73 years (average 51.6 years). The herniation was at L3/4 in 5 cases, L4/5 in 42 cases, and L5/S in 31 cases. The types of herniation were transligamentous extrusion in 55 cases and sequestration in 23 cases. The duration of disease before surgery was 13.3 ± 2.4 [mean ± standard deviation] weeks.

### MRI

MRI was performed using a 1.5 Tesla scanner before surgery (Magnetom Symphony, Siemens Healthcare). Unenhanced T1-weighted spin echo images (TR/TE, 540/12) and T2-weighted spin echo images (TR/TE, 2300/120) in sagittal and axial planes were obtained in all cases. The matrix size was 320 × 224 for both sagittal and axial images, with a field of view of 330 × 280 mm. The slice thickness and interslice gap were 4 and 1 mm, respectively, for both sagittal and axial slices. An experienced radiologist and an orthopedic surgeon, who were blinded to the study subjects, reviewed the existence of endplate degeneration on the basis of Modic's classification [22], disk degeneration on the basis of Pfirrmann's classification [23], vertebral corner defect of the posterior region, and the high-intensity zone (HIZ), which is believed to be correlated with the presence of severe annular disruption and low back pain [24]. Furthermore, to pinpoint the location and describe the extent of Modic changes, we classified the cases into six types using the original classification system, which was partially modified from previous reports [3,7]: anterior, middle, posterior, anterior and middle, middle and posterior, and all three areas combined.

### Sampling procedure and histopathological analysis

During surgery, all epidural tissues and disc material underneath the posterior longitudinal ligament (PLL) were removed. The intradiscal space was observed, and loose parts of the discs were also removed. The surgical specimens were fixed in a 10% formaldehyde solution and embedded in paraffin. Fragments with a diameter of ≥1 cm were cut with a knife in order to obtain a representative cross section. The materials were cut into 4-μm-thick slices using a microtome and stained with hematoxylin and eosin. At least three consecutive sections of the largest cross-sectional area were evaluated for each specimen. Two pathologists, who were blinded to the study subjects, examined the specimens and reported on the histological findings. They checked for the existence of cartilaginous endplates and bone tissues and examined the inflamed granulation tissue around the herniated mass. The following criteria were used to discriminate between the cartilaginous endplate and other tissues: the structure observed in the specimens is composed of hyaline cartilage, with an amorphous matrix and with chondrocyte-like cells exhibiting a clearly visible perinuclear halo. Although the cartilaginous endplate was easily demarcated, there was often no sharp dividing line between the nucleus pulposus and annulus fibrosus, especially in severely degenerated disc material. Because this study concentrates mainly on the relative percentage of cartilage, this factor can be neglected. The percentage of the area occupied by the cartilaginous endplates in the herniated material was also determined.

### Immunohistochemical staining

As for immunohistochemical staining, we conducted a histopathological analysis on 60 selected patients with inflamed granulation tissue that was adjacent to the herniated material. To evaluate the extent of neovascularization and macrophage infiltration of the herniated mass, the inflamed granulation tissue specimens that were observed around the herniated discs were analyzed by immunohistochemical staining for CD34 (mouse monoclonal antibody, QB-end-10, 1/50 dilution; Leica Biosystems, Newcastle upon Tyne, UK) and CD68 (mouse monoclonal antibody, KP1, 1/300 dilution; Dako, Glostrup, Denmark). Briefly, the specimens were deparaffinized in xylene and dehydrated in ethanol. After dehydration, the endogenous peroxidase was blocked by methanol containing 3% H_2_O_2_ for 30 min. For staining with the aforementioned antibody, the specimens were pretreated with citrate buffer (0.01 mol/L citric acid, pH 6.0) four times, and each pretreatment was carried out for 5 min at 100°C in a microwave oven. The specimens were incubated overnight with the primary antibody at 41°C, followed by staining with a streptavidin–biotin–peroxidase kit (Nichirei, Tokyo, Japan). They were then reacted in a 3,3′-diaminobenzidine, peroxytrichloride substrate solution, counterstained with hematoxylin, and mounted. The CD34-positive capillaries and CD68-positive cells were counted as the average of 20 randomly selected views from each specimen at 4 × 10 magnification [7].

### Statistical analysis

Chi-squared test and Fisher's exact test were used to calculate the differences in proportions for categorical variables. Group *t*-tests were used to compare the average number of CD34-positive capillaries and CD68-positive cells in the herniated tissue. The Bonferroni post-hoc adjustment for multiple comparisons was used to adjust the *p-*values for the total number of tests being conducted. Statistical analyses were performed using JMP^®^ version 11.0 (SAS Institute Inc., Cary, NC, USA). A *p*-value of <0.05 (with Bonferroni correction *p* < 0.0027 or *p* < 0.005) was considered statistically significant.

## Results

### MRI findings

Modic changes were observed in 26 (type 1, 7 cases; type 2, 17 cases; and type 3, 2 cases) of 78 patients (33%). The location of Modic changes was anterior in four cases, middle in five cases, posterior in three cases, anterior and middle in four cases, middle and posterior in five cases, and all three areas in five cases. With regard to disk degeneration, 17 cases were classified as grade 2, 23 cases as grade 3, 28 cases as grade 4, and 10 cases as grade 5. HIZ and vertebral corner defects were present in six (8%) and seven (9%) cases, respectively.

### Histopathological data

Cartilaginous endplates in herniated specimens were observed in 32 (41%) of 78 cases. Small bone fragments were found in three cases. The percentage of the area occupied by the cartilaginous endplates in herniated specimens was <25%, between 25% and 50%, and ≥50% in 20, 9, and 3 cases, respectively. Inflamed granulation tissue was observed in 60 cases (77%), found mostly around the periphery of the herniated tissue. The MRI and histopathological findings are summarized in Table 1.

**Table 1:** MRI and histopathological findings.

characteristics	Number of cases
Modic changes	
type	
none	52
1	7
2	17
3	2
Location	
anterior	4
middle	5
posterior	3
anterior + middle	4
middle + posterior	5
three areas	5
Disk degeneration	
Grade 2	17
Grade 3	23
Grade 4	28
Grade 5	10
High intensity zone	6
Vertebral corner defect	7
Cartilaginous endplate*	
< 25%	20
25%–50%	9
≥50%	3
inflamed granulation tissue	60

*The percentage of the area occupied by cartilaginous endplate.

### Relationship between clinical features, MRI findings, and histopathological findings

**Fig 1:**
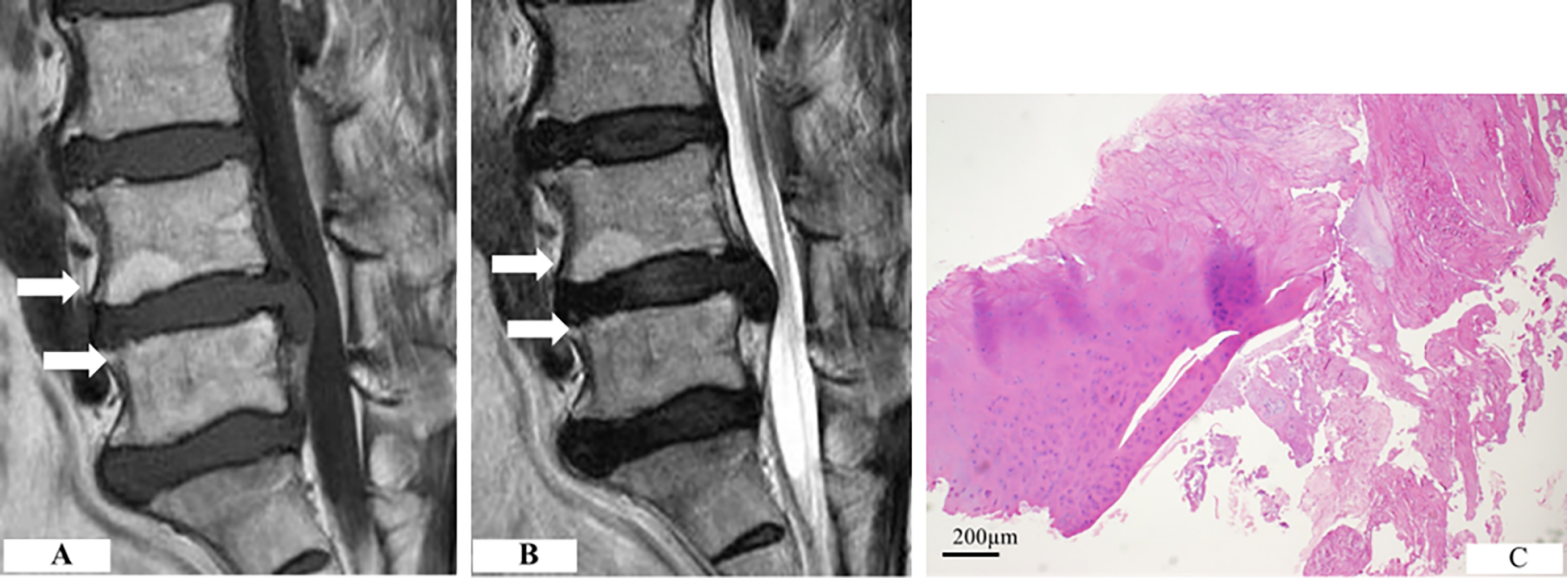
Cartilaginous herniation with Modic type 2 change. (A) The inferior endplate of L4 and the superior endplate of L5 show increased signal intensity on both T1-weighted scan. (B) The T2-weighted scan showed increased intensity in the same location. (C) Cartilaginous endplates were present in the herniated disc, along with the annulus fibrosus.

Cartilaginous endplates were more frequently observed in older patients (*p* = 0.0021; significance was set at *p* < 0.0027 with Bonferroni correction) (Table 2). No significant relationships were found between gender, level, types of herniation, and disease duration. Cartilaginous endplates were more frequently detected in patients with Modic changes than in those without Modic changes (65% versus 28%, *p* = 0.001; significance was set at *p* < 0.0027 with Bonferroni correction) (Table 2). Fig 1 shows cartilaginous herniation with Modic type 2 change in one typical patient. Of the patients with cartilaginous endplates with Modic changes, 4 had type 1 and 13 had type 2 Modic changes. Furthermore, patients with signal changes in the middle third of the vertebral endplate (14 of 19 cases, 74%) and in the extension of more than two areas (10 of 14 cases, 71%) showed a tendency for cartilaginous endplates (Table 3). No significant correlation between the presence of cartilaginous endplates and other MRI findings was detected. In addition, there was no correlation between inflamed granulation tissue and the findings of the clinical and MRI parameters.

**Table 2:** Relationship between clinical features, MRI findings, and histopathological findings.

	Cartilaginous endplate			Inflammatory granulation tissue		
Characteristics	+	−	*p* value	+	−	*p* value
Age (years)						
<50	11	32		33	10	
≥50	21	14	0.0021*	27	8	0.966
Gender						
Male	20	29		38	11	
Female	12	17	0.961	22	7	0.864
Herniation level †						
L4/5	14	28		35	7	
L5/S	16	15	0.116	24	7	0.525
Herniation type						
TE	20	35		43	12	
S	12	11	0.195	17	6	0.683
Disease duration (weeks)						
9–12	20	31		38	13	
13–16	12	15	0.655	22	5	0.486
Modic changes						
+	17	9		19	7	
−	15	38	0.001*	42	11	0.539
Disc degeneration						
Grade 2, 3	15	25		31	9	
Grade 4, 5	17	21	0.516	29	9	0,901
High intensity zone						
+	3	3		4	2	
−	29	43	0.641	56	16	0.534
Vertebral corner defect						
+	4	3		4	3	
−	28	43	0.363	56	15	0.192

TE; transligamentous extrusion, S; sequestration.

*Statistically significant (with Bonferroni correction, significance is set at *p* < 0.0027).

† *n* = 73.

**Table 3:** Relationship between cartilaginous endplate and detailed data of Modic changes.

	Cartilaginous endplate		
	+		−
Modic changes	≥25%*	<25%	
type			
1	2	2	3
2	5	8	4
3	0	0	2
Location			
anterior	0	1	3
middle †	2	2	1
posterior	1	1	1
anterior + middle † ††	0	2	2
middle + posterior † ††	2	2	1
three areas † ††	2	2	1

* The percentage of the area occupied by cartilaginous endplate.

† The presence of cartilaginous endplate in patients with Modic changes in the middle-third of the vertebral endplate (14/19, 74%).

†† The presence of cartilaginous endplate in patients with Modic changes in the extension of more than two areas (10/14, 71%).

### Immunohistochemical findings and relationship between cartilaginous endplates and inflammatory response

**Fig 2:**
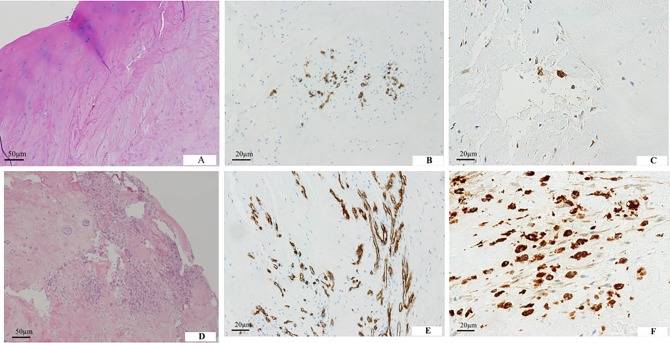
Composition of herniated tissues. (A) A herniated mass containing hyaline cartilage from the endplate. (B) CD34-positive capillaries are partly observed in the herniated specimen. (C) CD68-positive macrophages are less frequent. (D) Inflamed granulation tissue is observed in herniated specimens without cartilaginous endplates. (E) CD34-positive capillaries are distributed diffusely in the herniated specimen. (F) CD68-positive macrophages are abundant.

Immunohistochemical analysis was performed for 60 cases to evaluate inflamed granulation tissue adjacent to herniated material. Neovascularization was found in all herniated specimens, whereas the ratio of macrophage infiltration was 80% (48 cases). Fewer CD34-positive capillaries and CD68-positive cells were found in herniated specimens containing cartilaginous fragments than in those without cartilaginous fragments (*p* < 0.001, significance was set at *p* < 0.005 with Bonferroni correction) (Table 4). Fig 2 shows a histological specimen. There was no significant difference in the numbers of CD34-positive capillaries and CD 68-positive cells due to age (≥50 years versus <50 years), herniation type (transligamentous extrusion versus sequestration), disease duration (9–12 weeks versus 13–16 weeks) and the presence of Modic changes.

**Table 4:** The numbers of neovascularization and macrophage infiltrate in the herniated disc (*n* = 60).

	CD34 (+)	*p* value	CD68 (+)	*p* value
Age (years)				
<50 (n = 26)	13.74 ± 3.87		15.48 ± 3.82	
≥50 (n = 34)	14.52 ± 3.43	0.411	17.34 ± 3.57	0.056
Herniation type				
TE (n = 41)	12.68 ± 3.57		16.88 ± 3.72	
S (n = 19)	14.34 ± 3.28	0.102	16.16 ± 3.41	0.437
Disease duration (weeks)				
9–12 (n = 40)	12.55 ± 3.34		15.13 ± 3.83	
13–16 (n = 20)	14.34 ± 3.96	0.071	17.16 ± 3.54	0.052
Modic changes				
+ (n = 23)	12.74 ± 3.55		14.68 ± 3.29	
− (n = 37)	14.62 ± 3.67	0.06	16.97 ± 3.97	0.019
Cartilaginous endplate				
+ (n = 25)	8.24 ± 3.36		9.32 ± 3.29	
− (n = 35)	16.26 ± 3.67	< 0.001*	19.82 ± 3.67	<0.001*

mean ± SD.

TE; transligamentous extrusion, S; sequestration.

*Statistically significant (with Bonferroni correction, significance is set at *p* < 0.005).

A higher immunoreactivity to CD34 and CD68 was found in herniated discs <25% of whose area was occupied by cartilaginous endplates compared with discs whose area was occupied at 25% or more (*p* < 0.001) (Table 5).

**Table 5:** The numbers of neovascularization and macrophage infiltration in patients with LDH containing cartilaginous endplate (*n* = 25).

	CD34 (+)	*p* value	CD68 (+)	*p* value
The percentage of the area occupied by				
cartilaginous endplate				
<25 (n = 15)	11.58 ± 2.98		12.66 ± 2.96	
≥25 (n = 10)	4.32 ± 2.82	<0.001*	5.50 ± 2.50	<0.001*

mean ± SD.

*Statistically significant.

## Discussion

The morphology of LDH is heterogeneous and can include the nucleus pulposus, annulus fibrosus, or cartilaginous endplates. Previous studies have reported that LDH specimens in elderly populations frequently contain hard tissues, such as cartilaginous endplates and bone fragments [1,2,6,25]. Harada et al. revealed that cartilaginous endplate fragments comprised >70% of samples in patients in the seventh or eighth decade of life [2]. Rajasekaran et al. showed that a high proportion of Indian patients younger than 60 years had herniation with cartilage and bone fragments [4], indicating that avulsion-type LDH occurs in a substantial percentage of young patients. Some biomechanical studies reported the occurrence of endplate failure when loading occurred in pure compression/torsion and axial compression and flexion conditions [26,27]. Considering that fissuring of the outer annulus wall was less common in discs in patients younger than 30 years as previously reported [28], the failure of the endplate junction is more likely to occur in healthy discs subjected to overloading. This type of herniation tends to occur in the elderly with high frequency because of the weakness of endplate junction based on the degeneration of vertebral endplate structure with advancing age. In this study, we frequently observed cartilaginous endplate fragments in elderly patients, a result that is consistent with previous studies.

Signal intensity changes in the bone marrow adjacent to the vertebral endplates in degenerative spine disease are well known, and these changes were classified by Modic et al into three types [22]. Modic changes are especially associated with LDH that contains hyaline cartilage from vertebral endplates [3,7,8]. Schmid et al. demonstrated that vertebral endplate marrow signal intensity changes are indicative of cartilaginous endplates in the extruded herniated material, and Modic type 2 changes are predominant compared with type 1 changes [3]. With regard to the location of signal intensity changes, Modic changes in the middle third of the endplate, the region in which most avulsion occurs in the inner or transitional zone of the annulus–endplate interface, showed an association with cartilaginous herniation [1,3]. These results indicated that Modic changes act as a marker of cartilage herniation, that is, avulsion-type herniation accompanied by the rupture of the cartilaginous endplate from the vertebral body caused by endplate degeneration. These results were consistent with the present data, especially with the finding that Modic type 2 changes are predominant.

Many MRI studies have been conducted on spontaneous herniated disc resorption [9–12]. Histologically, granulation tissue surrounds the herniated mass and is characterized by inflammatory cell infiltration and newly formed vessels [15–18], involving several molecules, such as tumor necrosis factor-alpha, matrix-degrading enzymes, fibroblastic growth factor, and vascular endothelial growth factors [29–31]. A large, migrating herniation tends to regress more readily than a smaller herniation because penetration of the annulus fibrosus or PLL exposes disk material to the systemic circulation of the epidural space, thereby enhancing cellular inflammatory reactions [13,14]. To minimize differences in inflammatory reaction due to the type of herniation, our study was limited to analysis of patients with transligamentous and sequestration LDH perforating the PLL. Age has also been suggested as a factor influencing herniated disc resorption [11,32]. For example, studies reported that herniated discs in older patients contain less nucleus pulposus tissue and more annulus fibrosus tissue and cartilaginous endplate material, which probably inhibits inflammatory reactions, resulting in weaker immunological responses and angiogenesis in older patients [32,33]. Conversely, some MRI studies have suggested that the extent of herniated disc resorption is not correlated with age [9,34]. In this study, we observed no difference in neovascularization and macrophage infiltration due to age. We speculated that other factors, such as the size or type of herniation and disease duration, may be associated with herniated disc resorption in clinical studies.

Previous studies demonstrated that patients with greater percentages of cartilaginous endplates in the herniated material show significantly higher pain scores, with an increased duration of sciatica [5,6], probably due to disk hardness in endplate failure that can produce significant compression. Some studies showed a relationship between compression pressure and duration of the spinal nerve root conduction deficit [35,36]. However, Joe et al. reported that the composition of the extruded disk material is not associated with clinical symptoms [8]. In our study, the existence of a cartilaginous endplate in the herniated material had no correlation with the duration of disease before surgery. These results could be explained by the inflammatory effect of the nucleus pulposus with resulting pain; that is, the nucleus pulposus has been reported to induce physiologic changes in the nerve root regardless of the mechanical compression [37–40].

Several studies have reported that components of the herniated disc may influence spontaneous resorption [3,5–7]. An experimental animal study showed that neovascularization was observed in the implanted annulus fibrosus, but not in the cartilaginous endplate in the cornea, and that implanting a cartilaginous endplate together with the annulus fibrosus inhibits the growth of new vessels and inflammation [41]. Shan et al. reported that herniated discs with Modic changes are associated with cartilaginous herniation and that the herniated hyaline cartilage consequently shows less neovascularization and inflammatory infiltration for the extruded fragments [7]. Furthermore, our study showed a negative correlation between a greater area of cartilaginous endplate in the herniated disc and inflammatory response. The reason may be that the extruded annulus fibrosus and nucleus pulposus tissues swell rapidly, leading to a loss of proteoglycans that inhibit neovascularization [19,20]. However, the collagen network of hyaline cartilage severely limits swelling and proteoglycan loss [42], resulting in the inhibition of neovascularization and macrophage infiltration related to herniated disc resorption and persistent clinical symptoms. Our study showed that the inflammatory response in herniated discs may be influenced by the composition of herniated material, especially cartilaginous endplates, whose coverage of more area inhibits neovascularization and macrophage infiltration. To the best of our knowledge, this is the first study to evaluate the association between occupancy rate of cartilaginous endplates in the herniated material and the extent of inflammatory response using immunohistochemical analysis. These results suggest that the amount of cartilaginous endplate in herniated material affects herniated disc resorption, influencing the natural course of the disease.

Our study had a few limitations. First, the sample collection may have been subject to variance because the discectomy surgeries were performed by several surgeons. To minimize the variance, the herniated fragments were removed by the same surgical procedure. Second, the sample size was probably not large enough to yield substantial results. Thus, further studies with large sample sizes should be performed to confirm the present results. Third, we did not include clinical data, such as visual analog scale scores or functional evaluation scoring, and the association between the presence of cartilaginous endplates and sciatica and/or low back pain remains undetermined.

In conclusion, approximately half of the LDH cases with Modic changes contain some hyaline cartilage, with Modic type 2 changes being predominant. Furthermore, neovascularization and macrophage infiltration may be less frequent in cartilage herniation, especially if the amount of cartilage is high. Thus, a high percentage of cartilaginous endplate may result in the poor clinical course of patients with LDH.
